# Is machine learning the future for atrial fibrillation screening?

**DOI:** 10.1016/j.cvdhj.2022.04.001

**Published:** 2022-05-16

**Authors:** Pavidra Sivanandarajah, Huiyi Wu, Nikesh Bajaj, Sadia Khan, Fu Siong Ng

**Affiliations:** ∗ National Heart and Lung Institute, Imperial College London, London, United Kingdom; † Chelsea and Westminster NHS Foundation Trust, London, United Kingdom

**Keywords:** Atrial fibrillation, Machine learning, Screening, Artificial intelligence, Electronic health records

## Abstract

Atrial fibrillation (AF) is the most common arrhythmia and causes significant morbidity and mortality. Early identification of AF may lead to early treatment of AF and may thus prevent AF-related strokes and complications. However, there is no current formal, cost-effective strategy for population screening for AF. In this review, we give a brief overview of targeted screening for AF, AF risk score models used for screening and describe the different screening tools. We then go on to extensively discuss the potential applications of machine learning in AF screening.

**Key Findings**

• There may be long-term clinical benefits of atrial fibrillation (AF) screening suggested by the recent STROKESTOP study, but it is currently not cost-effective to conduct systematic AF screening.
• Machine learning can be applied to AF screening to help with AF risk prediction or to improve the automated diagnosis of AF using different rhythm monitoring modalities.
• Machine learning AF risk prediction can be used to predict incident, paroxysmal, and future AF. Prediction models have been developed using 12-lead electrocardiograms (ECGs), ambulatory ECGs, and electronic health records.
• Machine learning automated AF diagnosis has been achieved using a variety of rhythm modalities including 12-lead ECGs, ambulatory ECGs, and photoplethysmography.

## Introduction

Atrial fibrillation (AF) is the most common arrhythmia and causes significant morbidity and mortality, including strokes, heart failure, cognitive decline, depression, impaired quality of life, and hospitalizations.^1^ AF can be difficult to identify, as it can be paroxysmal and patients may be asymptomatic. Individuals with AF have a 5-fold increased risk of developing strokes, which can be reduced significantly (by up to two-thirds) with appropriate anticoagulation.^2^ However, currently, only opportunistic screening for AF is recommended by the European Society of Cardiology (ESC)^1^ and UK National Institute for Health and Care Excellence (NICE)^3^ and relies on patients presenting to general practices themselves, while in the United States, the US Preventive Services Task Force does not recommend AF screening.^4^ In this review, we will discuss how AF screening has previously been carried out and the current application of machine learning (ML) in AF screening.

## Recent studies on clinical outcomes of AF screening

Two recent studies reported the long-term clinical outcomes of AF screening. The STROKESTOP^5^ study performed in Sweden recruited 75- to 76-year-old participants between 2012 and 2014. They were randomized to a screening group or a control group (usual care). Individuals in the screening group were screened for AF over a 2-week period twice daily, using a single-lead electrocardiogram (ECG) recorder, Zenicor. There was a small net benefit of screening with regard to the primary endpoint, which was a composite of ischemic or hemorrhagic stroke, systemic embolism, bleeding requiring hospitalization, and all-cause death.

The LOOP study,^6^ carried out in neighboring Denmark, also looked at long-term clinical outcomes of AF screening. In this study, participants aged 70–90 years with at least 1 additional stroke risk factor were recruited. They were randomized in a 1:3 ratio to an implantable loop recorder monitoring group or control group (usual care). If a minimum of 6 minutes of AF was detected, anticoagulation was recommended. However, in this study, there was no reduction in stroke or systemic arterial embolism with screening.

The STROKESTOP study showed benefit for systematic AF screening, while the LOOP study did not. It has been postulated that a reason for this was that the background AF detection rate in the control group in LOOP was significantly higher than would be expected, thus reducing the benefit of AF screening. The duration of 6 minutes of AF requiring anticoagulation in LOOP may have also been too short a duration and perhaps anticoagulation should only be considered for longer periods of AF, although the minimum duration of AF that should require anticoagulation is still unknown.

## Targeted AF screening groups

Although there may be some clinical benefit in screening for AF, it is not cost-effective to conduct systematic screening, as only 1 case is identified after screening 145 individuals.^7^ Different screening studies have chosen specific groups of individuals for targeted screening to make systematic screening more cost-effective. The criteria for inclusion into these studies often have similarities, such as age, a certain CHA_2_DS_2_-VASc score or prior history of stroke.

It is known that the incidence of AF increases with age. Many screening studies choose age 65 as the minimum age to enter their screening study for AF. However, 1 meta-analysis of single–time point AF screening studies calculated the number needed to screen (NNS) to identify 1 case of AF in a population greater than 65 years old was still as high as 69, with the number rising to 83 to identify 1 treatable new AF case. This number increased the lower the age of the population chosen to screen.^8^

Another approach that has been used is to screen for AF based on the CHA_2_DS_2_-VASc score, which will be discussed in the next section, while others have used a prior history of stroke as an inclusion criterion for AF screening, as AF increases one’s risk of a stroke by 5-fold. However, despite targeting screening to the above groups, a cost-effective screening program with sufficiently low NNS has not been developed. Therefore, more work needs to be done to identify appropriate individuals for systematic targeted screening.

## Risk score models to target AF screening

Over the last 15 years, several clinical risk score models have been developed to predict the likelihood of the development of AF. They can provide a 5-year or 10-year prediction risk of AF, and they are listed in Table 1. Most of these were developed from data from particular population cohorts. The earlier risk scores were developed in the United States (US) from population cohorts, including FRAMINGHAM,^9^ ARIC,^10^ and CHARGE-AF,^11^ which have C-statistic values of 0.78, 0.78, and 0.765, respectively.

**Table 1:** Risk score models for atrial fibrillation detection

Risk score model	Factors	Internal validation	External validation	Prediction
FRAMINGHAM^9^	Age, blood pressure, hypertension, body mass index, PR interval, murmur, age of heart failure	C-statistic 0.78	C-statistic 0.65–0.73 Montefiore Medical Center (Bronx County): 0.734/0.724^12^ ARIC 0.68^10^ AGES 0.652^11^ Rotterdam 0.686^11^ CHARGE-AF 0.734^11^	10 -year risk of AF
ARIC^10^	Age, race, height, systolic blood pressure, hypertension medication, smoking, murmur, left atrial enlargement, LVH, diabetes mellitus, heart failure, age of coronary heart disease	C-statistic 0.78	-	10-year risk of AF
CHARGE-AF^11^	Age, race, height, weight, systolic blood pressure, diastolic blood pressure, murmur, hypertension medication, diabetes mellitus, heart failure, myocardial infarction, LVH on ECG, PR interval	C-statistic 0.765	C-statistic 0.66–0.81 Montefiore Medical Center (Bronx County): 0.707/0.691^12^ ARIC: 0.775 FHS: 0.75 EPIC Norfolk: 0.81/0.8 (2-fold overestimation of AF)^13^ MESA: 0.779^27^ Electronic health record only: 0.747/0.753^38^	5-year risk of AF
CHA_2_DS_2_-VASc	Congestive heart failure, hypertension, age, diabetes mellitus, vascular disease, stroke, sex	-	FHS: 0.71 ARIC: 0.66 Israeli database: 0.7440^14^ Chinese cohort: 0.687^17^ Korean cohort: 0.637^17^ Electronic health record only: 0.701/0.702^38^	Stroke risk
HATCH^39^	Heart failure, age, previous transient ischemic attack, chronic pulmonary obstructive disease, hypertension	-	Taiwan cohort: 0.7711^40^	Progression of paroxysmal AF to persistent AF
Suita^16^	Age, systolic hypertension, weight, alcohol intake, smoking, other arrhythmia, coronary artery disease, cardiac murmur	C-statistic 0.749	-	10-year risk of AF
Japanese simple risk score^15^	Age, sex, waist circumference, diastolic blood pressure, alcohol intake Added model: ECG variable	C-statistic 0.77 Added model C-statistic 0.79	-	7-year risk of AF
C2HEST^17^	Coronary artery disease, chronic obstructive pulmonary disease, hypertension, elderly, systolic heart failure, thyroid disease	C-statistic 0.75	Korean cohort: 0.65 Taiwan cohort: 0.7895^40^ Electronic health record only: 0.735/0.754^38^	AF risk
Taiwan AF score^18^	Age, sex, hypertension, heart failure, coronary artery disease, end-stage renal disease, alcoholism	AUROC 0.862 at 1 year, 0.755 at 16 years	-	1- to 16-year risk of AF
SAAFE^19^	Age, height, weight, congestive heart failure, coronary artery disease, chronic obstructive pulmonary disease, cardiac arrest, coronary artery stenting, stroke, diabetes, kidney transplant	C-statistic 0.785/0.804	ARIC 0.766	Incident AF risk
Electronic health record risk score^38^	Sex, age, race, smoking, height, weight, blood pressure, cardiovascular and cardiometabolic factors	C-statistic 0.77	C-index 0.808^41^	5-year risk of AF

AF = atrial fibrillation; AUROC = area under the receiver operating characteristic curve; ECG = electrocardiogram; LVH = left ventricular hypertrophy.

Following the development of a clinical risk score model from a particular population cohort, the model is usually evaluated on a different population cohort where the data are readily available. However, it has been recurrently found that the performance of a model is not as accurate as when performed on the original population cohort. The C-statistic value is often greater than 0.75 in the original population cohort and this drops below 0.75 when external validation of the model is performed. AGES, the Rotterdam study, and the EPIC Norfolk cohort are examples of population cohorts used for external validation. Recalibration or modifications of the model are required to maintain adequate performance.^12^^,^^13^ There are many differences in clinical variables between the cohorts that could affect the performance of the model, including median age, proportions of different ethnicities, and prevailing cardiovascular risk factors within the population cohort.

The CHA_2_DS_2_-VASc risk score is a score designed for stroke risk prediction in individuals with AF. Although CHA_2_DS_2_-VASc risk score is not formally validated for AF risk prediction, it has been used to identify individuals to screen for AF. It has been tested on a retrospective Israeli database to give an area under the receiver operating characteristic curve (AUROC) of 0.744 for prediction of new-onset AF.^14^ There are also a few clinical risk score models developed from Asian population cohorts, which include a Japanese simple risk score,^15^ Suita score,^16^ C2HEST,^17^ and the Taiwan AF score,^18^ which were developed more recently. There was a need for these, as previous risk scores have been developed from populations where Asian groups were underrepresented.

As listed in Table 1, there are now many clinical risk score models that have been developed for AF risk prediction. However, they are not applicable worldwide, as risk factors for AF will vary from population to population. One study compared 9 clinical risk score models on the ARIC cohort and found that only 5 risk scores were superior to using age alone. The best risk model, CHARGE-AF, was only able to identify 82% of the AF patients in the ARIC group.^19^ Many risk score models have attempted to incorporate examination findings, blood tests, ECG data, and echo data without improving the performance of the risk score model significantly. It is also difficult to obtain these types of data for individuals, as they are often missing and therefore affect the performance of the risk score model. These factors have limited the ability of these clinical risk score models to be used effectively for systematic AF screening.

## Screening tools to diagnose AF

There has been a recent growth in the number of technologies available for heart rhythm monitoring, which provides more opportunities for ambulatory monitoring, therefore increasing the likelihood of detecting AF. There are currently over 400 wearable devices available for monitoring and detection of AF.^1^ Clinically validated mobile health technologies include handheld or smartphone single-lead ECG recorders, smartwatch single-lead ECG recorders, photoplethysmography (PPG) smartwatches or smartphones, ECG patch monitors, blood pressure monitors, and external Holter monitors. Several studies have been performed for each modality showing that they are feasible to use for AF detection. The summaries of their results are listed in Table 2. A meta-analysis suggests that the screening modality does not influence the yield of screening^20^; therefore, with the appropriate targeted screening group, the use of any of these mobile health technologies could be very promising.

**Table 2:** Devices to detect and screen for atrial fibrillation

Rhythm modality	Reference	Results
Handheld/smartphone single-lead ECG recorder	William et al 2018,^42^Maputo et al 2020^43^	Algorithm sensitivity 0.966 Algorithm specificity 0.94 Physician review sensitivity 1 Physician review specificity 0.987
Handheld/smartphone single-lead ECG recorder	Vaes et al 2014^44^	Sensitivity 0.94 (0.87–0.98) Specificity 0.93 (0.85–0.97)
Smartwatch single-lead ECG recorder	Wasserlauf et al 2019^45^	Sensitivity 0.975 when compared to implantable cardiac monitor
Smartphone PPG	Brasier et al 2019^46^	Sensitivity 0.915 Specificity 0.996
Smartphone PPG	Proesmans et al 2019^47^	Sensitivity 0.96 Specificity 0.97
Smartphone PPG	Poh et al 2018^48^	Sensitivity 1 Specificity 0.996
Smartphone PPG (iPhone 4S)	McManus et al 2013^49^	Sensitivity 0.962 Specificity 0.975
Smartphone PPG (iPhone 4S)	Lee et al 2013^50^	Sensitivity 0.7461–0.9763 Specificity 0.9961–1
Smartwatch PPG	Bashar et al 2019^51^	Sensitivity 0.982 Specificity 0.974
Smartwatch PPG	Dörr et al 2019^52^	Sensitivity 0.937 (0.898–0.964) Specificity 0.982 (0.958–0.994)
Smartwatch PPG^51^	Bumgarner et al 2018^53^	Sensitivity 0.93 (0.86–0.99) Specificity 0.84 (0.73–0.95)
ECG patch monitor	Okubo et al 2021^54^	Sensitivity 0.98 Specificity 0.982
Blood pressure monitor	Wiesel et al 2009^55^	Sensitivity 0.95 Specificity 0.86

ECG = electrocardiogram; PPG = photoplethysmography.

## Machine learning

ML is one of the approaches of artificial intelligence, which itself is a broader concept of automation and prediction. Artificial intelligence has been proven beneficial in a wide range of applications, from trivial tasks for humans such as recognizing faces in an image or video to understanding large data to make decisions. There are various models for ML, which are shown in Table 3.

**Table 3:** Examples of machine learning and deep learning models

Machine learning model		Principle
Traditional models		
Logistic regression		Uses a linear combination of variable with logistic function for the classification
Decision trees / random forest		Uses the binary tree–based approach
Naïve Bayes		Uses probabilistic approach–based Bayes theorem and assumes that all the input variables are independent
Linear discriminant analysis		A dimensionality reduction method for classification. It looks for linear combinations of variables that best explain the data, and separates 2 or more classes of objects
Support vector machines		Uses a kernel-based hyperplane that separates the data points with maximum margin
K-nearest neighbor		Uses the distance (eg, Euclidian) of a given data point to K nearest data points using known data points (training set) to predict the class/value. It is based on assumption that data points in near proximity have similar values
Multilayer perceptron		Fully connected neural networks
Deep learning model		
Deep neural network		Originated from multilayer perceptron, uses different architectures and methods of weight sharing
Convolutional neural networks	ResNet	Uses shared weights to exploit translation invariance in convolution approach. ResNet uses skip connections, U-Net architecture is used to label pixels of an image, widely used for image segmentation
	U-Net	
Recurrent neural networks	GRU	Uses temporal dynamic behavior of data with recursive operations. It is efficient to carry the useful information over a long time using GRU and LSTM mechanisms. Transformers also process the temporal input data using attention mechanism, which allows it to handle very large temporal data.
	LSTM	
	Transformers	
Generative models	Auto-encoders, GANs	Generative models are based on approach to learn the distribution of given dataset, which can be used to reduce the dimension of datasets (eg, auto-encoders) or to generate new dataset (GANs)

GRU = gated recurrent units; LSTM = long short-term memory; GANs = generative adversarial networks.

**Figure 1:**
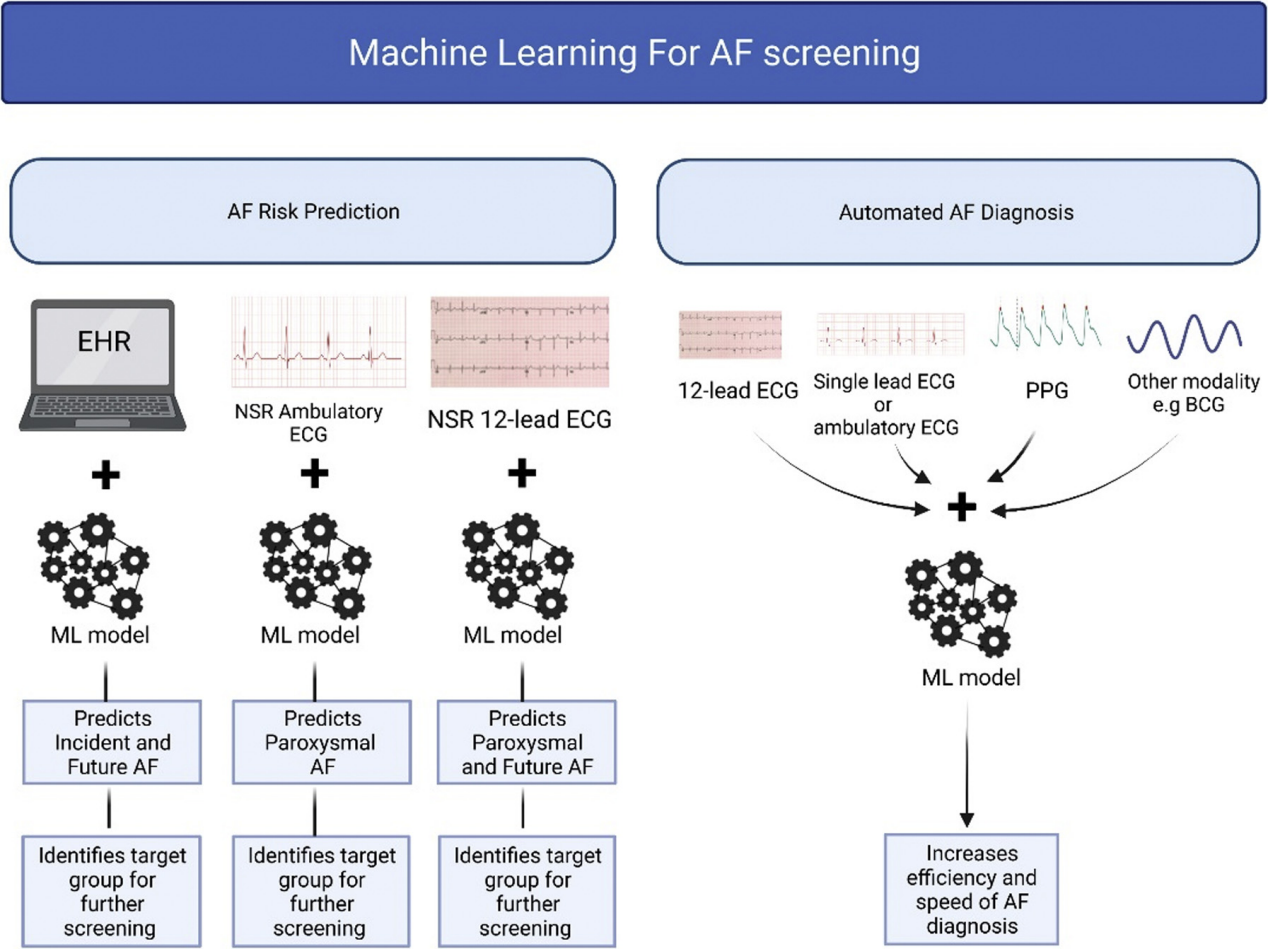
Summary of machine learning (ML) for atrial fibrillation (AF) screening. There are 2 main categories: AF risk prediction and automated AF diagnosis. AF risk prediction involves the application of ML to electronic health records, normal sinus rhythm ambulatory electrocardiograms (ECGs), and normal sinus rhythm 12-lead ECGs to identify the target group for further screening. For automated AF diagnosis, ML is applied to different rhythm monitoring modalities to increase efficiency and the speed of AF diagnosis. BCG = ballistocardiography; EHR = electronic health records; NSR = normal sinus rhythm; PPG = photoplethysmography.

ML algorithms can be applied to a wide range of healthcare data, which include and are not limited to mobile health technology data, electronic health records, biomedical imaging data, implanted electronic device data, biochemical data, and genomic data. This makes ML invaluable for automated detection of pathology or conditions, for risk prediction of future conditions, and for prediction of those who would benefit from specific treatment strategies. Some examples of the uses of ML for AF include prediction of recurrence of AF following AF ablation,^21^ improving cardiac activation mapping for AF,^22^ prediction of successful electrical cardioversion of AF,^23^ and AF screening. Thus far, supervised and unsupervised ML have been used for AF screening. ML can be applied to AF screening to help with 2 different tasks: (1) to identify the individuals with the highest risk for AF to target the screening to minimize the number needed to screen, and (2) to improve the automated diagnosis of AF using data from different rhythm monitoring modalities. We discuss both these applications below. Figure 1 shows a summary of ML in AF screening.

### Machine learning for AF risk prediction to target AF screening

Several studies have looked at the use of ML to predict future AF using 12-lead ECGs, electronic health records, or sinus rhythm ambulatory ECGs. These approaches can be used to help target any screening efforts to maximize the efficiency of screening and the number of new AF cases detected.

#### Machine learning for AF risk prediction using 12-lead ECGs

ML has been used to predict future or paroxysmal AF from sinus rhythm ECGs in 3 studies. The first one of these is from the Mayo Clinic.^24^ This study used 649,931 normal sinus rhythm ECGs from 180,922 patients collected over a 24-year period. Convolutional neural networks were used to create an algorithm that predicted from a sinus rhythm ECG the likelihood of paroxysmal AF. The AUROC was 0.87 and the sensitivity was 0.79. The algorithm was further validated on the Mayo Clinic Olmsted Study of Aging cohort.

Another study^25^ looked at predicting current AF and future AF from a sinus rhythm ECG with 2 separate models. The study used 12,863 sinus rhythm ECGs without a history of structural heart disease from the Shinken Database 2010–2017. The predictive models were based on a random forest algorithm. This study also explored ECG parameters that could affect predictive capability using logistic regression and Spearman coefficient of correlation. It identified that the P wave, QRS, and ST-T segment were similar in their parameter importance to the model. The C-statistic for prediction of current paroxysmal AF was 0.913 and the C-statistic for prediction of future AF was 0.991.

The third study that predicts the future risk of AF from the ECG is from Geisinger.^26^ This study used 1.6 million resting 12-lead ECG traces from 430,000 patients collected over a 35-year period for training, and then using new-onset AF at 1 year for testing. This model achieved sensitivity of 0.69, specificity of 0.81, and AUROC of 0.85. In addition, this model has been tested on patients who experienced an AF-related stroke within 3 years of the indexed ECG, and it predicted new-onset AF with accuracy of 62% in this subgroup of patients. This suggests that the model could also help predict future AF-related strokes. However, all these models have yet to be used prospectively.

#### Machine learning for AF risk prediction using ambulatory ECGs

ML has been applied to sinus rhythm segments of ambulatory ECGs to predict the presence of paroxysmal AF. Supplemental Table 1 summarizes these studies. The preceding sinus rhythm segments can be used to predict the probability of an incoming episode of paroxysmal AF. There are 2 approaches for this, which are using heart rate variability features or premature atrial complexes (PACs) features. The initial approach was using PACs, as it is found that there is an increase in PACs preceding a paroxysmal AF episode. However, recent approaches have involved using heart rate variability features, which include time domains and frequency domains. The accuracy of models developed in the last year for predicting paroxysmal AF have been above 0.9.

#### Machine learning for AF risk prediction using electronic health records

It is not possible for everyone to have an ECG or a physiological signal recording to predict the risk of AF. Another approach is to use healthcare data from electronic healthcare records to identify possible patients that may have AF. This would reduce the number of individuals needed to screen. The use of ML in combination with clinical risk scoring shows potential for reducing the NNS and improving the effectiveness of AF screening.

Random survival forests have been used in the Multi-Ethnic Study of Atherosclerosis (MESA) cohort^27^ to identify highly predictive variables for the prediction of AF. These variables included coronary calcium score, carotid measurements, blood test results, ECG measurements, and echocardiographic measurements. The variables were combined to the CHARGE-AF enriched score (CHARGE-AF score with NT-proBNP) to create a new score, which gave a C-statistic of only 0.806. The C-statistic for the CHARGE-AF enriched score was 0.804. In this case, the use of ML was not beneficial in improving AF risk prediction.

There have been 4 further AF screening studies that have applied an ML screening tool previously using data from healthcare databases. They all compared different ML models to find the most optimum model for AF screening and are summarized in Supplemental Table 2.

A team at the University of Colorado developed their own ML model for 6-month risk prediction of AF using electronic health records.^28^ They compared the use of different ML models, including Naïve Bayes, logistic regression, random forest, shallow and deep neural networks, and gradient-boosted machine, and found that single-layer neural networks and random oversampling was the best model. The sensitivity was 75.2% and the specificity was 84.9% The AUROC was 0.8. However, they found that a simple logistic regression model using known clinical risk factors performed nearly as well as their ML model. This study, however, did not take into account the time-varying effects of the variables.

Another group,^29^ using secondary care data from Japanese databases, also developed their own ML methods for the identification of nonvalvular AF patients. The ML models used included deep learning with lasso regression. Stacking models of multiple single classifiers were also used, including lasso regression, ridge regression, support vector machine, random forest, deep learning, AdaBoost, and gradient boosting. The AUROC was greater than 0.8 for each individual model and, for stacking models, was as high as 0.9. The sensitivity varied between 0.09 and 0.84. The specificity varied between 0.79 and 0.99 for each model, while for stacking models they were 0.8 or above. The best model was a stacking model that included deep learning (neural networks) with lasso regression.

The most recent study^30^ to develop an ML model to apply to electronic healthcare records compared only different logistic regression models. The healthcare records used were derived from the Indiana Network for Patient Care. The best model, the UNAFIED model, used 10 variables and had a C-statistic of 0.8061 following validation phase. The model was implemented prospectively as part of a 6-week pilot study on the Eskenazi Health Information Systems. However, the prospective patients identified did not undergo further monitoring, screening, or intervention to confirm if they had AF.

Our group recently helped to develop and optimize another ML algorithm to identify individuals at highest risk of AF. The algorithm was trained by using a dataset from a large United Kingdom primary care database called UK Clinical Practice Research Datalink.^31^ It was created using time-varying neural networks and the published AF risk score models, Framingham, ARIC, and CHARGE-AF. Baseline ML models were compared initially, which included neural networks, lasso regression, random forest, and support vector machine. Time-varying covariates were then considered and added. The AUROC was 0.827 and the NNS was 9. We then evaluated and validated this ML algorithm on the separate DISCOVER database retrospectively.^32^ The DISCOVER database contains primary care data from over 2.5 million people living in northwest London. The AUROC increased to 0.87 and the NNS remained at 9. We also evaluated and validated the ML algorithm on our secondary care database retrospectively.^33^ The sensitivity was 0.5 and the specificity was 0.95 with an NNS of 5. It showed an additional 5444 individuals who had AF that were not identified by algorithm based on their primary care data alone. However, as with the other ML algorithms, this has not yet been tested prospectively.

### Machine learning to improve automated AF diagnosis

There are a wide range of rhythm monitoring modalities including ECGs used for AF detection. Another application of ML in AF screening is to improve the accuracy of automated AF diagnosis using different rhythm monitoring modalities, which could have the potential to increase the numbers of individuals screened and allow quicker population or mass screening. It could mean that a physician is no longer required to interpret the result of the rhythm monitoring modality. The use of ML with different rhythm monitoring modalities is discussed below and is summarized in Supplemental Tables 3–6.

#### Machine learning to improve automated AF diagnosis using 12-lead ECGs

Supplemental Table 3 shows different studies that have developed ML models to detect AF from a 12-lead ECG. The models typically used are neural networks, as these allow unknown features of the ECG to be used in the decision process to determine if the ECG demonstrates AF. There was only 1 study that used a vector machine instead. Neural networks do not necessarily require the ECG data to have undergone feature extraction and selection.

There were high accuracy and AUC (>0.98) values for models differentiating between AF and sinus rhythm. However, the performance of the models for AF detection reduced when the models were also developed to differentiate other rhythms too.

#### Machine learning to improve automated AF diagnosis using photoplethysmography

There have been multiple studies that have tested ML algorithms to detect AF from PPG signals. PPG involves reading pulse pressure signals resulting from the propagation of blood pressure pulses along arterial blood vessels.^34^ It can be measured peripherally. Detection of irregularities in pulse pressure signals aids in the diagnosis of AF. The ML studies using PPG for AF diagnosis are shown in Supplemental Table 4. The ML models used were neural networks, logistic regression, random forests, and support vector machines. When the models were compared, neural networks were found to be superior. The accuracy, specificity, and sensitivity of these models were above 0.90. The features that ML models review include pulse interval features and pulse amplitude features, which can make distinguishing AF from ectopic beats difficult, although 1 study has used deep learning classifiers to overcome this. PPG is more susceptible to noise and artifact than a 12-lead ECG; however, DeepBeat (another deep learning model) has also overcome this. PPG showing AF still needs a confirmatory ECG to confirm the AF diagnosis. However, the use of ambulatory monitoring PPG could reduce the number of individuals requiring a confirmatory ECG to diagnose AF.

#### Machine learning to improve automated AF diagnosis using single-lead or ambulatory ECGs

Supplemental Table 5 lists a large number of studies involving ML algorithms to identify AF from single-lead or ambulatory ECGs. PhysioNet Challenge 2017, AF prediction, MIT-BIH AF, and MIT-BIH Arrhythmia databases are readily available to the public and were used by several studies for training and testing their models. The easy accessibility of a large database of single-lead ECGs has made it possible for many ML models to be developed compared with 12-lead ECGs. A wide range of models were used. When measured, accuracy, sensitivity, and specificity tended to be above 0.85. However, when F1 scores were measured, these would vary from 0.782 to 0.92. In the last year, feature importance interpretability and real-time detection have been researched. Their aims have been to explore explainable artificial intelligence and improve AF diagnosis time.

#### Machine learning to improve automated AF diagnosis using other diagnostic modalities

Supplemental Table 6 shows 3 studies showing ML algorithms applied to other forms of monitoring for AF. One method is ballistocardiography, which provides waves based on ejection of blood from the heart to the great vessels. Other methods were using arterial pulse waveforms and inertia measurements using a gyroscope and accelerometer on a smartphone. Different models were used for the different methods. Again, accuracy, sensitivity, and specificity for these models tended to be above 0.9.

### Limitations of studies applying machine learning for AF screening

One general limitation of the ML studies is the imbalance in data between the size of the AF cohort and the non-AF cohort, as the non-AF cohort is usually considerably larger. However, this was not the case for the Indiana Network owing to the population containing a higher proportion of older patients. To deal with the imbalances in data, resampling methods such as oversampling or undersampling have been used.

A limitation regarding the development of these ML models from electronic patient records is that the non-AF cohort may include patients who have undiagnosed AF and therefore are not labeled as having AF when training the ML algorithm. It is also not clear if, in the AF cohort, there may be patients who are also misdiagnosed with AF. A prospective, interventional study actively diagnosing AF patients could address this issue.

With regard to automated rhythm diagnosis, in the presence of multiple ectopic beats (premature atrial complexes or ventricular complexes), there can be a high risk of false-positive AF findings owing to rhythm irregularity.^35^ This can consequently impact accuracy of ML for automated AF diagnosis. Memory-stored templates of ectopic beats can help to overcome misinterpretation of ectopic beats for AF. For PPG, tachograms, Lorenz plots,^36^ or Poincare plots^37^ can be used to aid in improving accurate AF diagnosis.

The impact of missing data on the efficacy of an ML algorithm for AF screening is also unclear. There is limited transparency about how the processes of ML lead to their outputs. Although there are inherent limitations to the accuracy of the ML algorithms, in theory, they will most likely outperform current practice of opportunistic screening for AF.

The ideal ML algorithm for AF risk prediction would use electronic health records in combination with 12-lead ECGs. However, the availability of 12-lead ECG data from electronic health records and in an appropriate form limits our ability to develop such an algorithm.

## Conclusion

There have been many developments in the use of ML to improve AF screening. These ML algorithms have either been used to identify the individuals at highest risk of AF to better target the screening or to facilitate more accurate automated AF diagnosis during the screening process. Most ML algorithms have been developed and tested on retrospective data, but there needs to be more work to test them prospectively and then to assess their impact on real-world data.

## Appendix Supplementary data

Supplemental Tables 1–6 and Supplemental References
