# Supplementary material for: Is machine learning the future for atrial fibrillation screening?
Source: Cardiovasc Digit Health J. 2022 May 16;3(3):136–45. doi: 10.1016/j.cvdhj.2022.04.001 (PMC9204790; doi:10.1016/j.cvdhj.2022.04.001)
Supplement: Supplemental Tables 1–6 and Supplemental References [file mmc1.docx]

**Supplementary File**

**Table 1: Use of Machine learning for AF risk prediction using ambulatory ECGs**

| **Reference** | **Model** | **Data/cohort Design** | **Specific points** | **Results** |
| --- | --- | --- | --- | --- |
| Ganapathy et al 2021^1^ | Dynamic Symbol Assignment and Random Forest/ SVM | AFPDB and AF Termination challenge database | - | Accuracy 0.998 |
| Bashar et al 2021^2^ | SVM/ Random Forest | Testing: MIMIC III  Training: AFPDB |  | Accuracy: 0.9  10 mins before episode- Accuracy: 0.8 |
| Wang et al 2021^3^ | Quantum particle optimisation SVM | Fuzhou University + Fujian Hospital, AFDB, AFPDB |  | Accuracy 0.91 |
| Parsi et al 2021^4^ | Linear kernel SVM | AFPDB | Poincare representation | Accuracy 0.98  Sensitivity 0.988  Specificity 0.967 |
| Erdenebayar et al 2019^5^ | CNN | MIT-BIH AF Database+ MIT-BIH NSR Database + Paroxysmal AF prediction challenge database |  | Sensitivity:0.986  Specificity:0.987  Accuracy:0.987 |
| Sutton et al 2019^6^ | Random Forest | 30 min ECG recordings,  50 controls, 25 NSR recordings from PAF patients, 25 PAF episodes | - | Sensitivity for PAF: 1 |
| Ebrahimzadeh et al 2018^7^ | Multilayer perceptron vs K-nearest neighbour vs SVM vs mixture | AF Prediction Database | - | For PAF  Sensitivity: 1  Specificity: 0.955 |
| Narin et al 2018^8^ | K-nearest neighbour | AF Prediction Database | - | For PAF:  Sensitivity: 0.84  Specificity: 0.84  Accuracy: 0.848 |
| Boon et al 2018^9^ | SVM | AF Prediction Database | - | For PAF  Sensitivity: 0.868  Specificity: 0.887  Accuracy: 0.877 |
| Mohebbi et al 2012^10^ | SVM | AF Prediction Database |  | For PAF  Sensitivity: 0.96  Specificity: 0.931  Accuracy: 0.877 |

**Table 2: Use of Machine Learning for AF risk prediction using electronic health records**

| **Reference** | **Model** | **Data/cohort Design** | **Results** |
| --- | --- | --- | --- |
| Tiwari et al 2020^11^ | Single layer NN + random oversampling | Database of 3 hospitals in Colorado | AUROC 0.8  Sensitivity 0.752  Specificity 0.849 |
| Suzuki et al 2019^12^ | Deep neural networks and lasso regression | Japanese Claims Database | AUC 0.9  Sensitivity 0.82  Specificity 0.82 |
| Grout et al 2021^13^ | Logistic regression | Indiana Network for Patient Care Database, proof of concept:  Eskenazi Health | C-statistic 0.8061 |
| Hill et al 2019^14^, Sekelj et al 2020^15^ | Time varying neural networks | UK CPRD, validated on North West London database | AUROC 0.827/0.87 |

**Table 3: Studies applying Machine Learning to 12-lead ECGs to improve Automated AF Diagnosis**

| **Reference** | **Model** | **Data/cohort Design** | **Specific points** | **Results** |
| --- | --- | --- | --- | --- |
| Ribeiro et al 2020^16^ | Deep neural networks (residual network) | > 2 million ECGs from Telehealth Network of Minas Gerais- Brazil | ECG classification- AF, sinus tachycardia, sinus bradycardia, LBBB, RBBB and 1^st^ degree AVB  Performance compared to emergency resident, cardio resident, medical student | For AF detections: sensitivity: 0.769, specificity: 1.0  PPV 1.0, F1 score: 0.87 |
| Jo et al 2020^17^ | Neural backed ensemble tree | Trained with Sejong dataset: 128 399 ECGs  Tested with PTB XL, Chapman, PhysioNet | - | AUC using 12 lead ECG: 0.997-0.999 |
| Zhang et al 2020^18^ | CNN | 277807 ECGs from First Affiliated Hospital of Nanjing Medical University 2018-19 | - | Classify normal rhythm/AF  Accuracy for AF:0.9827 |
| Kong et al 2019^19^ | Integrated radial basis function + relevance vector machine (sparser than SVM) | - | 1056 AF patients  904 health patients | Classification rate: 0.9816 using channel II |

**Table 4: Studies applying Machine Learning to PPG data to improve Automated AF Diagnosis**

| **Reference** | **Model** | **Data/cohort Design** | **Specific points** | **Results** |
| --- | --- | --- | --- | --- |
| Valiaho et al 2021^20^ | Logistic regression | 359 x 5 min PPG recordings |  | AUC 0.982/0.993  Sensitivity 0.951/0.964  Specificity  0.937/0.963 |
| Jacobsen et al 2020^21^ | Deep neural networks vs normalised root mean square of successive differences | 102 inpatients admitted with AF (107 enrolled) | Each patient worn armband(PPG) and ECG holter | DNN- best  AUC 0.98, Sensitivity 0.96, Specificity 0.99 |
| Han et al 2020^22^ | Random Forests |  | Poincare plots- PPG Smartwatch, classification PVC, PAC, AF | 10- fold cross validation for AF  Accuracy: 0.95  Sensitivity: 0.92  Specificity: 0.96 |
| Eerikainen et al 2020^23^ | Random Forests | 40 patients | PPG with accelerometer and 24hr holter used. Classification included AF, atrial flutter, and SR, SVPB, VOB | For AF detection:  Sensitivity: 0.976  Specificity: 0.982 |
| Kwon et al 2020^24^ | Convoluted neural networks vs support vector machines +/- root mean square of successive differences and Shannon entropy | 100 participants with persistent AF for elective DCCV | PPG ring type wearable | CNN-best  Sensitivity: 0.9896  Specificity: 0.9434 |
| Torres-Soto et al 2020^25^ | Multitask CNN vs Random Forest | Stanford Uni dataset of pts undergoing exercise stress test or elective cardioversion (training, validation, testing)  Prospective ambulatory monitoring n=15 | PPG signals  Wrist watch based PPG signals | AF detection in ambulatory group  Sensitivity: 0.98  Specificity: 0.99 |
| Pereira et al 2019^26^ | CNN (ResNet18) vs SVM | 2 cohorts (3764 vs 13) of ICU pts (1 included stroke patients) | 30 second PPG converted to 2D images | CNN- best  For AF detection  Accuracy 0.977  Sensitivity 0.9835  Specificity0.9742 |
| Kwon et al 2019^27^ | 1d CNN and RNN vs SVM | 75 patients undergoing elective DCCV for persistent AF |  | 1D CNN  Accuracy: 0.9758 Sensitivity: 0.9932 Specificity: 0.9585  RNN  Accuracy: 0.9715  Sensitivity: 0.9827 Specificity 0.9604 |
| Wasserlauf et al 2019^28^ | CNN | Training: 7500 Alivercor users  Testing: 26 PAF patients with ICM | Smartwatch PPG with ICM | AF episode sensitivity: 0.975 |
| Poh et al 2018^29^ | CNN vs 6 other models | 3 databases | PPG converted to spectrogram  Min 17s of recording | AUC 0.997  Sensitivity: 0.952  Specificity:0.99 |

**Table 5: Studies applying Machine Learning to single lead ECGs/ ambulatory ECGs/ long term ECG for Automated AF Diagnosis**

| **Reference** | **Model** | **Data/cohort Design** | **Specific points** | **Results** |
| --- | --- | --- | --- | --- |
| Tanigarchi et al 2021^30^ | CNN + gradient weighted class activation mapping | 57 273 Holter ECGs | - | Accuracy 0.953  Sensitivity 0.971  Specificity 0.945 |
| Ilbeigipour et al 2021^31^ | Random forest + Apache Structured Streaming (real time detection) | MIT- BIH Database | Classification: RBBB  Normal  AF | AUC 0.862  Sensitivity 0.838  Accuracy 0.838  Specificity 0.975 |
| Rouhi at al 2021^32^ | Shapley Additive explanation (Feature importance interpretability) + Random Forest/ SVM | PhysioNet |  | F -score 0.746 |
| Michel et al 2021^33^ | γ Metric (filter approach feature selection)  and SVM | MIT-BIH NSR, MIT- BIH NSR RR, MIT-BIH AF, Validation: 105 patients 24hr ECG recordings |  | Accuracy >0.99 |
| Chen et al 2021^34^ | Multi-feature extraction and CNN | MIT-BIH Database |  | Accuracy 0.9892  Sensitivity 0.9719  Specificity 0.9704 |
| Yue et al 2021^35^ | Gaussian kernel SVM with frequency slice wavelet transform | MIT-BIH AF database |  | Accuracy 0.934 |
| Lown et al 2020^36^ | SVM | Trained with MIT-BIH AF Database + MIT-BIH Arrhythmia Database  Tested on 415 participants at 3 GP practices in Hampshire | Participants wore Polar H7 | Sensitivity: 1  Specificity:0.976 |
| Chocron et al 2020^37^ | Random Forest | Trained and tested on 3 databases  Evaluated on a sleep health heart study database | Single lead ECG signals with some 2 channel ECGs  Sleep database contained OSA and non-OSA. | Classification of prominent AF or not  Sensitivity:0.98  Specificity:0.99  Identification of AF not affected by mod-severe OSA |
| Mousavi et al 2020^38^ | Bidirectional recurrent neural networks (HAN-ECG3) | 2 datasets- 1- 10h long term ECG (MIT-BIH AF Database) and 2^nd^- 60s single lead ECG recording | Single lead ECG | AF classification based on dataset  Sensitivity:0.9053,0.8602  Specificity:0.7954, 0.9862  AUC:0.8946, 0.9846 |
| Yazid et al 2020^39^ | Support vector machines | ECG recordings sampled from MIT-BIH AF Database + MIT-BIH Arrhythmia Database | Single lead ECG signals were converted to local binary pattern codes before putting them into model | AF classification:  Best values with 60s recordings  Sensitivity: 0.9911  Specificity: 0.9929 |
| Zhang et al 2020^40^ | Compression sensing- CNN | MIT-BIH AF Database | 10hr ECGs underwent compression sensing before putting into CNN  1 second only needed to compress 24hr of data  Compared to SVM model | F1 score only given based on compression ratio  0.8817- 0.9625 |
| Buscema et al 2020^41^ | Deep neural networks (Supervised contractive map) | ECG recordings sampled from MIT-BIH AF Database + MIT-BIH Arrhythmia Database | ECG data converted into R-R interval distances | Accuracy: 0.95 |
| Abdelazez et al 2020^42^ | CNN with compressive sensing | Long term AF Database + MIT-BIH AF Database |  | AUC 0.75 -0.87 |
| Kisohara et al 2020^43^ | CNN | 24hr ECG obtained from 2 databases including ALLSTAR database | R-R intervals of 24hr ECG were converted into Lorenz plots before ML | Based on window length of beats  AUC 0.98-0.987  Sensitivity:0.975-0.998  Specificity: 0.928-0.978 |
| Bashar et al  2021^44^ | SVM, Autoregressive model, KNN, RF, coefficient of sample entropy | Trained with MIMIC III database  Tested on wearable armband ECG data | 2min ECGs converted into Poincare plots( based on differences in HR) | Best model: SVM  Sensitivity:0.9899  Specificity: 0.9999  Accuracy: 0.9998 |
| Bashar et al 2021^45^ | SVM, RF, KNN | Trained with MIMIC III database  Validated on wearable armband ECG data + AFPDB database | 2min ECGs converted into Poincare plots( based on differences in HR) | Classification of AF vs PAC/PVC  Best model: SVM  Sensitivity:0.9899  Specificity: 0.9518  Accuracy: 0.9745 |
| Bashar et al 2020^46^ | SVM, discriminant analysis, KNN | MIMIC III database | - | Sensitivity: 1  Specificity: 0.98  Accuracy: 0.9899 |
| Maruccini et al 2020^47^ | ANN | Single lead ECG databases | - | AUC: 0.908 for testing |
| Lai et al 2020^48^ | CNN | Patients wore patch based four lead ECG with 12 lead holter for 24hrs | 4 CNN models compared | Sensitivity:0.931  Specificity: 0.934  Accuracy: 0.931 |
| Ghosh et al 2020^49^ | DNN (Hierarchical Extreme Learning Machine) | MIT-BIH AF Database + MIT-BIH Arrhythmia Database | - | Sensitivity:0.9986  Specificity: 1  Accuracy: 0.9993 |
| Huang et al 2020^50^ | CNN | MIT-BIH AF Database + MIT-BIH NSR Database | ECG signals were filtered with Daubechies wavelet | Filtered ECG  Sensitivity:0.9971  Specificity: 0.9866  Accuracy: 0.9923  Non-Filtered ECG Sensitivity:0.9931  Specificity: 0.9903  Accuracy: 0.9918 |
| Hseiah et al 2020^51^ | 1D CNN | Single lead ECG Database | Length normalisation algorithm to get equal length recordings | F1 score 0.782 |
| Shi et al 2019^52^ | Multiple input deep neural networks | MIT-BIH AF Database + MIT-BIH Arrhythmia Database | - | Sensitivity:0.9746  Specificity: 0.811  Accuracy: 0.874 |
| Oster et al 2020^53^ | SVM+ CNN | Trained on PhysioNet/Cinc 2017  Evaluated on UK Biobank | Single lead ECGs  4 lead ECGs | Sensitivity: 0.75  PPV: 0.975  F1 score: 0.848 |
| Czabanski et al 2020^54^ | Lagrangian SVM | MIT-BIH AF Database | - | Sensitivity: 0.9894  Specificity:0.9880  Accuracy: 0.9886 |
| Shao et al 2020^55^ | CatBoost classifier (gradient boosting- decision trees) | AFDB 2017 + MIT-BIH AF Database | ECG path- Android App- Cloud Server | F1 score: 0.92 |
| Hannun et al 2019^56^ | End to end deep neural networks | Training and testing on single lead ECG from Zio monitor (91 232 + 328)  Validation on PhysioNet/ Cinc 2017 | Classification for 12 rhythm classes | For atrial fibrillation and flutter,  Sensitivity: 0.861  F1 score: 0.801/0.831 |
| Jiang et al 2019^57^ | CNN |  | ECG to BCG | Sensitivity: 0.983  Specificity:0.933  Accuracy: 0.958 |
| Kalidas et al 2019^58^ | Markov models and random forests | MIT-BIH AF Database + MIT-BIH Arrhythmia Database | Long term ECG | Sensitivity: 0.974/0.963  Specificity:0.986/0.97 |
| Wu et al 2019^59^ | CNN | MIT-BIH AF Database + MIT-BIH Arrhythmia Database + MIT BIH Malignant ventricular arrhythmia database + MIT-BIH NSR Database+ Long term AF Database + MIT-BIH Noise Stress Test Database | Single lead ECG (continuous wavelet transformation- wavelet coefficient) | Sensitivity: 0.9756  Specificity: 0.9919  Accuracy: 0.9756  AUC: 0.9983 |
| Ivanovic et al 2019^60^ | DNN (CNN +RNN) | 1097 30- second ECGs from Clinical Centre of Serbia | Also discriminate between SR, AF, and atrial flutter | For AF detection,  Sensitivity: 0.9420 |
| Faust et al 2018^61^ | RNN + Long short-term memory | MIT-BIH AF Database | Uses R-R intervals | Sensitivity: 0.9987  Specificity: 0.9961  Accuracy: 0.9977 |
| Gliner et al 2018^62^ | SVM, NN | PhysioNet Challenge 2017 | Normal rhythm, noise, AF, other | For SVM – AF  F1 score: 0.8 |
| Shao et al 2018^63^ | AdaBoosted decision tree ensemble | PhysioNet Challenge 2017 | Normal rhythm, noise, AF, other | F1 score- AF: 0.82 |
| Xiong et al 2018^64^ | CNN | PhysioNet Challenge 2017 | Sinus rhythm, AF, other rhythms | F1 score classification: 0.82 |
| He et al 2018^65^ | CNN | MIT-BIH AF Database | Denoise- segmentation- CWT | Sensitivity: 0.9941  Specificity: 0.9891  Accuracy: 0.9923 |
| Parvaneh et al 2018^66^ | CNN | PhysioNet Challenge 2017 | Normal rh^59^ythm, noise, AF, other | F1 score- AF: 0.82 |
| Khamis et al 2018^67^ | 10 NN | PhysioNet Challenge 2017 | Normal rhythm, noise, AF, other, Uses R-R intervals and PQRST morphology | F1 score- AF: 0.79 |
| Hernandez et al 2018^68^ | ANN | PhysioNet Challenge 2017 |  | F1 score- AF: 0.6161 |
| Liu et al 2018^69^ | SVM | PhysioNet Challenge 2017 |  | F1 score- AF: 0.7856 |
| Sadr et al 2018^70^ | Quadratic neural networks | PhysioNet Challenge 2017 |  | F1 score- AF: 0.75 |
| Goodfellow et al 2018^71^ | XGBoost | PhysioNet Challenge 2017 |  | F1 score- AF: 0.8156 |
| Xia et al 2018^72^ | DCNN | MIT-BIH AF Database | Short term Fournier transform + Stationery wavelet transformation | Sensitivity: 0.9834  Specificity: 0.9824  Accuracy: 0.9829 |
| Nuryani et 2017^73^ | SVM | MIT-BIH AF Database |  | Sensitivity :0.9847  Specificity: 0.9784 |
| Lemkadda-m et al 2017^74^ | SVM vs KNN | MIT-BIH Arrhythmia Database | PVC, normal, VT, VB, AF | For AF  Accuracy: 0.78 |
| Andersen et al 2017^75^ | SVM | MIT-BIH AF Database | R peaks | Sensitivity: 0.9681  Specificity:0.962  AUC: 0.99 |
| Kennedy et al 2016^76^ | Random Forests vs KNN | MIT-BIH AF Database + MIT-BIH Arrhythmia Database + + MIT-BIH NSR Database+ Long term AF Database + MIT-BIH SVA Database + SPIT + THEW |  | Sensitivity: 0.928  Specificity: 0.983 |

**Table 6: Studies applying Machine Learning to other diagnostic modalities for Automated AF Diagnosis**

| **Reference** | **Model** | **Data/cohort Design** | **Specific points** | **Results** |
| --- | --- | --- | --- | --- |
| Zalabarria et al 2020^77^ | ANN | 744 Arterial pulse waveforms from BP devices with ECGs- clinics in Auckland NZ | - | 20- fold cross validation  For AF diagnosis  Sensitivity:0.9753  Specificity:0.9013 |
| Yu et al 2019^78^ | Ensemble methods (Bagged trees- base learners) | 12 AF patients during sleep | BCG collected from electromechanical film | Sensitivity: 0.97  Specificity: 0.891  Accuracy: 0.944 |
| Lahdenoja et al 2018^79^ | Kernel SVM | 16 AF patients and 23 controls | Inertia measurement using gyroscope and accelerometer on smartphone | Sensitivity: 0.938  Specificity: 1  Accuracy: 0.974 |

References:

1. Ganapathy N, Baumgärtel D, Deserno TM. Automatic Detection of Atrial Fibrillation in ECG Using Co-Occurrence Patterns of Dynamic Symbol Assignment and Machine Learning. *Sensors*. 2021;21(10). doi:10.3390/s21103542

2. Bashar SK, Ding EY, Walkey AJ, McManus DD, Chon KH. Atrial Fibrillation Prediction from Critically Ill Sepsis Patients. *Biosensors*. 2021;11(8). doi:10.3390/bios11080269

3. Wang LH, Yan ZH, Yang YT, et al. A Classification and Prediction Hybrid Model Construction with the IQPSO-SVM Algorithm for Atrial Fibrillation Arrhythmia. *Sensors*. 2021;21(15). doi:10.3390/s21155222

4. Parsi A, Glavin M, Jones E, Byrne D. Prediction of paroxysmal atrial fibrillation using new heart rate variability features. *Computers in Biology and Medicine*. 2021;133:104367. doi:10.1016/j.compbiomed.2021.104367

5. Erdenebayar U, Kim H, Park JU, Kang D, Lee KJ. Automatic Prediction of Atrial Fibrillation Based on Convolutional Neural Network Using a Short-term Normal Electrocardiogram Signal. *J Korean Med Sci*. 2019;34(7). https://doi.org/10.3346/jkms.2019.34.e64

6. J. R. Sutton, R. Mahajan, O. Akbilgic, R. Kamaleswaran. PhysOnline: An Open Source Machine Learning Pipeline for Real-Time Analysis of Streaming Physiological Waveform. *IEEE Journal of Biomedical and Health Informatics*. 2019;23(1):59-65. doi:10.1109/JBHI.2018.2832610

7. Ebrahimzadeh E, Kalantari M, Joulani M, Shahraki RS, Fayaz F, Ahmadi F. Prediction of paroxysmal Atrial Fibrillation: A machine learning based approach using combined feature vector and mixture of expert classification on HRV signal. *Computer Methods and Programs in Biomedicine*. 2018;165:53-67. doi:10.1016/j.cmpb.2018.07.014

8. Narin A, Isler Y, Ozer M, Perc M. Early prediction of paroxysmal atrial fibrillation based on short-term heart rate variability. *Physica A: Statistical Mechanics and its Applications*. 2018;509:56-65. doi:10.1016/j.physa.2018.06.022

9. Boon KH, Khalil-Hani M, Malarvili M. Paroxysmal atrial fibrillation prediction based on HRV analysis and non-dominated sorting genetic algorithm III. *Computer Methods and Programs in Biomedicine*. 2018;153:171-184. doi:10.1016/j.cmpb.2017.10.012

10. Mohebbi M, Ghassemian H. Prediction of paroxysmal atrial fibrillation based on non-linear analysis and spectrum and bispectrum features of the heart rate variability signal. *Computer Methods and Programs in Biomedicine*. 2012;105(1):40-49. doi:10.1016/j.cmpb.2010.07.011

11. Tiwari DK. A retrospective observational research to assess the incidence of acute ischemic stroke in hospitalized atrial fibrillation patients with anticoagulation interruptions. *J Cardiovasc Dis Res*. 2021;12(4):934-938. doi:10.31838/jcdr.2021.12.04.112

12. Suzuki R, Katada J, Ramagopalan S, McDonald L. Potential of machine learning methods to identify patients with nonvalvular atrial fibrillation. *Future Cardiology*. 2019;16(1):43-52. doi:10.2217/fca-2019-0056

13. Grout RW, Hui SL, Imler TD, et al. Development, validation, and proof-of-concept implementation of a two-year risk prediction model for undiagnosed atrial fibrillation using common electronic health data (UNAFIED). *BMC Medical Informatics and Decision Making*. 2021;21(1):112. doi:10.1186/s12911-021-01482-1

14. Hill NR, Ayoubkhani D, McEwan P, et al. Predicting atrial fibrillation in primary care using machine learning. *PLoS One*. 2019;14(11):e0224582-e0224582. doi:10.1371/journal.pone.0224582

15. Sekelj S, Sandler B, Johnston E, et al. Detecting undiagnosed atrial fibrillation in UK primary care: Validation of a machine learning prediction algorithm in a retrospective cohort study. *Eur J Prev Cardiolog*. Published online August 13, 2020:2047487320942338. doi:10.1177/2047487320942338

16. Ribeiro AH, Ribeiro MH, Paixão GMM, et al. Automatic diagnosis of the 12-lead ECG using a deep neural network. *Nature Communications*. 2020;11(1):1760. doi:10.1038/s41467-020-15432-4

17. Jo YY, Cho Y, Lee SY, et al. Explainable artificial intelligence to detect atrial fibrillation using electrocardiogram. *International Journal of Cardiology*. 2021;328:104-110. doi:10.1016/j.ijcard.2020.11.053

18. Zhang X, Gu K, Miao S, et al. Automated detection of cardiovascular disease by electrocardiogram signal analysis: a deep learning system. *Cardiovasc Diagn Ther*. 2020;10(2):227-235. doi:10.21037/cdt.2019.12.10

19. Kong D, Zhu J, Wu S, Duan C, Lu L, Chen D. A novel IRBF-RVM model for diagnosis of atrial fibrillation. *Comput Methods Programs Biomed*. 2019;177:183-192. doi:10.1016/j.cmpb.2019.05.028

20. Väliaho ES, Kuoppa P, Lipponen JA, et al. Wrist Band Photoplethysmography Autocorrelation Analysis Enables Detection of Atrial Fibrillation Without Pulse Detection. *Frontiers in Physiology*. 2021;12:576. doi:10.3389/fphys.2021.654555

21. Jacobsen M, Dembek TA, Ziakos AP, et al. Reliable Detection of Atrial Fibrillation with a Medical Wearable during Inpatient Conditions. *Sensors*. 2020;20(19). doi:10.3390/s20195517

22. Han D, Bashar SK, Zieneddin F, et al. Digital Image Processing Features of Smartwatch Photoplethysmography for Cardiac Arrhythmia Detection. *Annu Int Conf IEEE Eng Med Biol Soc*. 2020;2020:4071-4074. doi:10.1109/EMBC44109.2020.9176142

23. L. M. Eerikäinen, A. G. Bonomi, F. Schipper, et al. Detecting Atrial Fibrillation and Atrial Flutter in Daily Life Using Photoplethysmography Data. *IEEE Journal of Biomedical and Health Informatics*. 2020;24(6):1610-1618. doi:10.1109/JBHI.2019.2950574

24. Kwon S, Hong J, Choi EK, et al. Detection of Atrial Fibrillation Using a Ring-Type Wearable Device (CardioTracker) and Deep Learning Analysis of Photoplethysmography Signals: Prospective Observational Proof-of-Concept Study. *J Med Internet Res*. 2020;22(5):e16443-e16443. doi:10.2196/16443

25. Torres-Soto J, Ashley EA. Multi-task deep learning for cardiac rhythm detection in wearable devices. *npj Digital Medicine*. 2020;3(1):116. doi:10.1038/s41746-020-00320-4

26. Pereira T, Ding C, Gadhoumi K, et al. Deep learning approaches for plethysmography signal quality assessment in the presence of atrial fibrillation. *Physiological Measurement*. 2019;40(12):125002. doi:10.1088/1361-6579/ab5b84

27. Kwon S, Hong J, Choi EK, et al. Deep Learning Approaches to Detect Atrial Fibrillation Using Photoplethysmographic Signals: Algorithms Development Study. *JMIR Mhealth Uhealth*. 2019;7(6):e12770. doi:10.2196/12770

28. Wasserlauf J, You C, Patel R, Valys A, Albert D, Passman R. Smartwatch Performance for the Detection and Quantification of Atrial Fibrillation. *Circulation: Arrhythmia and Electrophysiology*. 2019;12(6):e006834. doi:10.1161/CIRCEP.118.006834

29. Poh MZ, Poh YC, Chan PH, et al. Diagnostic assessment of a deep learning system for detecting atrial fibrillation in pulse waveforms. *Heart*. 2018;104(23):1921. doi:10.1136/heartjnl-2018-313147

30. Taniguchi H, Takata T, Takechi M, et al. Explainable Artificial Intelligence Model for Diagnosis of Atrial Fibrillation Using Holter Electrocardiogram Waveforms. *International Heart Journal*. 2021;62(3):534-539. doi:10.1536/ihj.21-094

31. Ilbeigipour S, Albadvi A, Akhondzadeh Noughabi E. Real-Time Heart Arrhythmia Detection Using Apache Spark Structured Streaming. Chen XJ, ed. *Journal of Healthcare Engineering*. 2021;2021:6624829. doi:10.1155/2021/6624829

32. Rouhi R, Clausel M, Oster J, Lauer F. An Interpretable Hand-Crafted Feature-Based Model for Atrial Fibrillation Detection. *Frontiers in Physiology*. 2021;12:581. doi:10.3389/fphys.2021.657304

33. Michel P, Ngo N, Pons JF, Delliaux S, Giorgi R. A filter approach for feature selection in classification: application to automatic atrial fibrillation detection in electrocardiogram recordings. *BMC Medical Informatics and Decision Making*. 2021;21(4):130. doi:10.1186/s12911-021-01427-8

34. Chen X, Cheng Z, Wang S, et al. Atrial fibrillation detection based on multi-feature extraction and convolutional neural network for processing ECG signals. *Computer Methods and Programs in Biomedicine*. 2021;202:106009. doi:10.1016/j.cmpb.2021.106009

35. Yue Y, Chen C, Liu P, Xing Y, Zhou X. Automatic Detection of Short-Term Atrial Fibrillation Segments Based on Frequency Slice Wavelet Transform and Machine Learning Techniques. *Sensors*. 2021;21(16). doi:10.3390/s21165302

36. Lown M, Brown M, Brown C, et al. Machine learning detection of Atrial Fibrillation using wearable technology. *PLOS ONE*. 2020;15(1):e0227401. doi:10.1371/journal.pone.0227401

37. Chocron A, Efraim R, Mandel F, et al. Machine learning for nocturnal mass diagnosis of atrial fibrillation in a population at risk of sleep-disordered breathing. *Physiological Measurement*. 2020;41(10):104001. doi:10.1088/1361-6579/abb8bf

38. Mousavi S, Afghah F, Acharya UR. HAN-ECG: An interpretable atrial fibrillation detection model using hierarchical attention networks. *Computers in Biology and Medicine*. 2020;127:104057. doi:10.1016/j.compbiomed.2020.104057

39. Yazid M, Abdur Rahman M. Variable step dynamic threshold local binary pattern for classification of atrial fibrillation. *Artificial Intelligence in Medicine*. 2020;108:101932. doi:10.1016/j.artmed.2020.101932

40. Zhang H, Dong Z, Gao J, Lu P, Wang Z. Automatic screening method for atrial fibrillation based on lossy compression of the electrocardiogram signal. *Physiological Measurement*. 2020;41(7):075005. doi:10.1088/1361-6579/ab979f

41. Buscema PM, Grossi E, Massini G, Breda M, Della Torre F. Computer Aided Diagnosis for atrial fibrillation based on new artificial adaptive systems. *Computer Methods and Programs in Biomedicine*. 2020;191:105401. doi:10.1016/j.cmpb.2020.105401

42. M. Abdelazez, S. Rajan, A. D. C. Chan. Transfer Learning for Detection of Atrial Fibrillation in Deterministic Compressive Sensed ECG. In: *2020 42nd Annual International Conference of the IEEE Engineering in Medicine & Biology Society (EMBC)*. ; 2020:5398-5401. doi:10.1109/EMBC44109.2020.9175813

43. Kisohara M, Masuda Y, Yuda E, Ueda N, Hayano J. Optimal length of R–R interval segment window for Lorenz plot detection of paroxysmal atrial fibrillation by machine learning. *BioMedical Engineering OnLine*. 2020;19(1):49. doi:10.1186/s12938-020-00795-y

44. Bashar SK, Hossain MB, Lázaro J, et al. Feasibility of atrial fibrillation detection from a novel wearable armband device. *Cardiovasc Digit Health J*. 2021;2(3):179-191. doi:10.1016/j.cvdhj.2021.05.004

45. S. K. Bashar, D. Han, F. Zieneddin, et al. Novel Density Poincaré Plot Based Machine Learning Method to Detect Atrial Fibrillation From Premature Atrial/Ventricular Contractions. *IEEE Transactions on Biomedical Engineering*. 2021;68(2):448-460. doi:10.1109/TBME.2020.3004310

46. Bashar SK, Han D, Hajeb-Mohammadalipour S, et al. Atrial Fibrillation Detection from Wrist Photoplethysmography Signals Using Smartwatches. *Scientific Reports*. 2019;9(1):15054. doi:10.1038/s41598-019-49092-2

47. Marinucci D, Sbrollini A, Marcantoni I, Morettini M, Swenne CA, Burattini L. Artificial Neural Network for Atrial Fibrillation Identification in Portable Devices. *Sensors*. 2020;20(12). doi:10.3390/s20123570

48. D. Lai, Y. Bu, Y. Su, X. Zhang, C. -S. Ma. Non-Standardized Patch-Based ECG Lead Together With Deep Learning Based Algorithm for Automatic Screening of Atrial Fibrillation. *IEEE Journal of Biomedical and Health Informatics*. 2020;24(6):1569-1578. doi:10.1109/JBHI.2020.2980454

49. Ghosh SK, Tripathy RK, Paternina MRA, Arrieta JJ, Zamora-Mendez A, Naik GR. Detection of Atrial Fibrillation from Single Lead ECG Signal Using Multirate Cosine Filter Bank and Deep Neural Network. *Journal of Medical Systems*. 2020;44(6):114. doi:10.1007/s10916-020-01565-y

50. Huang ML, Wu YS. Classification of atrial fibrillation and normal sinus rhythm based on convolutional neural network. *Biomedical Engineering Letters*. 2020;10(2):183-193. doi:10.1007/s13534-020-00146-9

51. Hsieh CH, Li YS, Hwang BJ, Hsiao CH. Detection of Atrial Fibrillation Using 1D Convolutional Neural Network. *Sensors (Basel)*. 2020;20(7):2136. doi:10.3390/s20072136

52. Shi H, Wang H, Qin C, Zhao L, Liu C. An incremental learning system for atrial fibrillation detection based on transfer learning and active learning. *Computer Methods and Programs in Biomedicine*. 2020;187:105219. doi:10.1016/j.cmpb.2019.105219

53. Oster J, Hopewell JC, Ziberna K, et al. Identification of patients with atrial fibrillation: a big data exploratory analysis of the UK Biobank. *Physiological Measurement*. 2020;41(2):025001. doi:10.1088/1361-6579/ab6f9a

54. Czabanski R, Horoba K, Wrobel J, et al. Detection of Atrial Fibrillation Episodes in Long-Term Heart Rhythm Signals Using a Support Vector Machine. *Sensors*. 2020;20(3). doi:10.3390/s20030765

55. Shao M, Zhou Z, Bin G, Bai Y, Wu S. A Wearable Electrocardiogram Telemonitoring System for Atrial Fibrillation Detection. *Sensors*. 2020;20(3). doi:10.3390/s20030606

56. Hannun AY, Rajpurkar P, Haghpanahi M, et al. Cardiologist-level arrhythmia detection and classification in ambulatory electrocardiograms using a deep neural network. *Nature Medicine*. 2019;25(1):65-69. doi:10.1038/s41591-018-0268-3

57. Jiang F, Xu J, Lu Z, et al. A Transfer Learning Approach to Detect Paroxysmal Atrial Fibrillation Automatically Based on Ballistocardiogram Signal. *Journal of Medical Imaging and Health Informatics*. 2019;9:1943-1949. doi:10.1166/jmihi.2019.2819

58. Kalidas V, Tamil LS. Detection of atrial fibrillation using discrete-state Markov models and Random Forests. *Computers in Biology and Medicine*. 2019;113:103386. doi:10.1016/j.compbiomed.2019.103386

59. Z. Wu, X. Feng, C. Yang. A Deep Learning Method to Detect Atrial Fibrillation Based on Continuous Wavelet Transform. In: *2019 41st Annual International Conference of the IEEE Engineering in Medicine and Biology Society (EMBC)*. ; 2019:1908-1912. doi:10.1109/EMBC.2019.8856834

60. M. D. Ivanovic, V. Atanasoski, A. Shvilkin, L. Hadzievski, A. Maluckov. Deep Learning Approach for Highly Specific Atrial Fibrillation and Flutter Detection based on RR Intervals. In: *2019 41st Annual International Conference of the IEEE Engineering in Medicine and Biology Society (EMBC)*. ; 2019:1780-1783. doi:10.1109/EMBC.2019.8856806

61. Faust O, Shenfield A, Kareem M, San TR, Fujita H, Acharya UR. Automated detection of atrial fibrillation using long short-term memory network with RR interval signals. *Computers in Biology and Medicine*. 2018;102:327-335. doi:10.1016/j.compbiomed.2018.07.001

62. Gliner V, Yaniv Y. An SVM approach for identifying atrial fibrillation. *Physiological Measurement*. 2018;39(9):094007. doi:10.1088/1361-6579/aadf49

63. Shao M, Bin G, Wu S, Bin G, Huang J, Zhou Z. Detection of atrial fibrillation from ECG recordings using decision tree ensemble with multi-level features. *Physiological Measurement*. 2018;39(9):094008. doi:10.1088/1361-6579/aadf48

64. Xiong Z, Nash MP, Cheng E, Fedorov VV, Stiles MK, Zhao J. ECG signal classification for the detection of cardiac arrhythmias using a convolutional recurrent neural network. *Physiological Measurement*. 2018;39(9):094006. doi:10.1088/1361-6579/aad9ed

65. He R, Wang K, Zhao N, et al. Automatic Detection of Atrial Fibrillation Based on Continuous Wavelet Transform and 2D Convolutional Neural Networks. *Frontiers in Physiology*. 2018;9:1206. doi:10.3389/fphys.2018.01206

66. Parvaneh S, Rubin J, Rahman A, Conroy B, Babaeizadeh S. Analyzing single-lead short ECG recordings using dense convolutional neural networks and feature-based post-processing to detect atrial fibrillation. *Physiological Measurement*. 2018;39(8):084003. doi:10.1088/1361-6579/aad5bd

67. H. Khamis, J. Chen, J. Stephen Redmond, N. H. Lovell. Detection of Atrial Fibrillation from RR Intervals and PQRST Morphology using a Neural Network Ensemble. In: *2018 40th Annual International Conference of the IEEE Engineering in Medicine and Biology Society (EMBC)*. ; 2018:5998-6001. doi:10.1109/EMBC.2018.8513496

68. F. Hernández, D. Méndez, L. Amado, M. Altuve. Atrial Fibrillation Detection in Short Single Lead ECG Recordings Using Wavelet Transform and Artificial Neural Networks. In: *2018 40th Annual International Conference of the IEEE Engineering in Medicine and Biology Society (EMBC)*. ; 2018:5982-5985. doi:10.1109/EMBC.2018.8513562

69. Liu N, Sun M, Wang L, Zhou W, Dang H, Zhou X. A support vector machine approach for AF classification from a short single-lead ECG recording. *Physiological Measurement*. 2018;39(6):064004. doi:10.1088/1361-6579/aac7aa

70. Sadr N, Jayawardhana M, Pham TT, Tang R, Balaei AT, de Chazal P. A low-complexity algorithm for detection of atrial fibrillation using an ECG. *Physiological Measurement*. 2018;39(6):064003. doi:10.1088/1361-6579/aac76c

71. Goodfellow SD, Goodwin A, Greer R, Laussen PC, Mazwi M, Eytan D. Atrial fibrillation classification using step-by-step machine learning. *Biomedical Physics & Engineering Express*. 2018;4(4):045005. doi:10.1088/2057-1976/aabef4

72. Xia Y, Wulan N, Wang K, Zhang H. Detecting atrial fibrillation by deep convolutional neural networks. *Computers in Biology and Medicine*. 2018;93:84-92. doi:10.1016/j.compbiomed.2017.12.007

73. Nuryani N, Harjito B, Yahya I, Solikhah M, Chai R, Lestari A. Atrial fibrillation detection using support vector machine and electrocardiographic descriptive statistics. *International Journal of Biomedical Engineering and Technology*. 2017;24:225. doi:10.1504/IJBET.2017.085140

74. Lemkaddem A, Proença M, Delgado R, et al. *An Autonomous Medical Monitoring System: Validation on Arrhythmia Detection*. Vol 2017.; 2017:4556. doi:10.1109/EMBC.2017.8037869

75. Andersen R, Poulsen E, Puthusserypady S. *A Novel Approach for Automatic Detection of Atrial Fibrillation Based on Inter Beat Intervals and Support Vector Machine*. Vol 2017.; 2017:2042. doi:10.1109/EMBC.2017.8037253

76. Kennedy A, Finlay DD, Guldenring D, Bond RR, Moran K, McLaughlin J. Automated detection of atrial fibrillation using R-R intervals and multivariate-based classification. *Journal of Electrocardiology*. 2016;49(6):871-876. doi:10.1016/j.jelectrocard.2016.07.033

77. Zalabarria U, Irigoyen E, Lowe A. Diagnosis of atrial fibrillation based on arterial pulse wave foot point detection using artificial neural networks. *Computer Methods and Programs in Biomedicine*. 2020;197:105681. doi:10.1016/j.cmpb.2020.105681

78. B. Yu, B. Zhang, L. Xu, P. Fang, J. Hu. Automatic Detection of Atrial Fibrillation from Ballistocardiogram (BCG) Using Wavelet Features and Machine Learning. In: *2019 41st Annual International Conference of the IEEE Engineering in Medicine and Biology Society (EMBC)*. ; 2019:4322-4325. doi:10.1109/EMBC.2019.8857059

79. O. Lahdenoja, T. Hurnanen, Z. Iftikhar, et al. Atrial Fibrillation Detection via Accelerometer and Gyroscope of a Smartphone. *IEEE Journal of Biomedical and Health Informatics*. 2018;22(1):108-118. doi:10.1109/JBHI.2017.2688473
